# Identification of ceftriaxone-resistant *Neisseria gonorrhoeae* FC428 clone and isolates harboring a novel mosaic* penA* gene in Chengdu in 2019–2020

**DOI:** 10.1186/s12941-023-00614-x

**Published:** 2023-08-17

**Authors:** Di Wang, Youwei Wang, Yamei Li, Leshan Xiu, Gang Yong, Yang Yang, Weiming Gu, Junping Peng

**Affiliations:** 1 NHC Key Laboratory of Systems Biology of Pathogens, Institute of Pathogen Biology, Chinese Academy of Medical Sciences & Peking Union Medical College, Beijing, China; 2 Key Laboratory of Respiratory Disease Pathogenomics, Chinese Academy of Medical Sciences & Peking Union Medical College, Beijing, China; 3 Institute of Dermatology and Venereology, Sichuan Academy of Medical Sciences & Sichuan Provincial People’s Hospital, Chengdu, China; 4 Department of Laboratory Medicine, The First Affiliated Hospital of USTC, Division of Life Sciences and Medicine, University of Science and Technology of China, Hefei, China; 5 School of Global Health, Chinese Center for Tropical Diseases Research, Shanghai Jiao Tong University School of Medicine, Shanghai, China; 6 Shanghai Skin Disease Hospital, Tongji University School of Medicine, Shanghai, China

**Keywords:** *Neisseria gonorrhoeae*, Ceftriaxone resistance, FC428, A8806, Homologous recombination

## Abstract

**Background:**

Antimicrobial resistance in gonorrhea has become a growing global public health burden. *Neisseria gonorrhoeae* isolates with resistance to ceftriaxone, the last remaining first-line option, represent an emerging threat of untreatable gonorrhea.

**Methods:**

A total of ten ceftriaxone-resistant *N. gonorrhoeae* FC428 isolates and two isolates harboring a novel mosaic *penA*-232.001 allele from 160 gonococcal isolates in Chengdu in 2019–2020 was described in the present study. Multilocus sequence typing (MLST) and *N. gonorrhoeae* sequence typing for antimicrobial resistance (NG-STAR) were performed to characterize the isolates. Whole genome sequencing and maximum-likelihood method were performed to infer how the genetic phylogenetic tree of these isolates looks like. Recombination analysis was performed using the RDP4 software. This study was registered in the Chinese Clinical Trial Registry (ChiCTR2100048771, registration date: 20210716).

**Results:**

The genetic phylogeny showed that the ten FC428 isolates sporadically clustered into different phylogenetic clades, suggesting different introductions and local transmission of FC428. Two isolates showed close genetic relatedness to ceftriaxone-resistant clone A8806, which was only reported from Australia in 2013. Homologous recombination events were detected in *penA* between *Neisseria gonorrhoeae* and commensal *Neisseria* species (*N. perflava* and *N. polysaccharea*), providing evidence of commensal *Neisseria* species might serve as reservoirs of ceftriaxone resistance-mediating *penA* sequences in clinical gonococcal strains.

**Conclusions:**

Our results demonstrate further dissemination of FC428 in China and resurgence risks of sporadic ceftriaxone-resistant A8806 to become the next clone to spread.

**Supplementary Information:**

The online version contains supplementary material available at 10.1186/s12941-023-00614-x.

## Background

*Neisseria gonorrhoeae* isolates with resistance to ceftriaxone, the last remaining first-line option, represent an emerging threat of untreatable gonorrhea [1]. The ceftriaxone-resistant FC428 was first discovered in Japan in 2015 [2], first reported in China in 2016 [3] and subsequently worldwide [4–7]. Recently, FC428-related isolates harboring *penA*-60.001 allele have been reported in the USA in 2019 [8], in southern China in 2021[9], in Sweden in 2022 [10], and etc. High-dose ceftriaxone therapy is effective in uncomplicated gonorrhea [11, 12], however, it brings a threat to public health, the environment and antimicrobial resistance. The emergence of ceftriaxone-resistant FC428 isolates caused widespread concerns.

Chengdu is the capital city of Sichuan and the major cultural and economic center in southwest China, which is one of the sentinel surveillance sites of the China Gonococcal Resistance Surveillance Program (China-GRSP) [13, 14]. The general incidence of gonorrhea (3.56/100 000) is low in Sichuan [15], but ceftriaxone resistance has raised great concerns since FC428 was first reported in 2018 in Chengdu. *penA*-60.001, a feature of FC428 carrying mutations A311V and T483S, is associated with cephalosporin resistance. Both mutations were present in ceftriaxone-resistant isolates A8806 (*penA*-64.001) [16], H041 (*penA*-37.001) [17] and GU140106 (*penA*-59.001) [18]. The Australian A8806 displayed a ceftriaxone minimum inhibitory concentration (MIC) of 0.5 mg/L, which has not been reported elsewhere [16]. Two of the indexed isolates in this research harboring a novel mosaic *penA*-232.001 allele showed close genetic relatedness to A8806, indicating the potential risk of resurgence and further transmission of A8806.

## Methods

A total of 160 gonococcal isolates were collected, specifically 92 in 2019 and 68 in 2020, respectively, which were obtained from outpatients with gonorrhea who attended the Sichuan Provincial People’s Hospital in Chengdu during 2019 to 2020. All isolates were screened from the Multicentre Clinical Evaluation project of resistance of *N. gonorrhoeae* using high-resolution melting assays previous published [19, 20]. The MICs of the antimicrobials against the 12 isolates were determined using the agar dilution method [21] (Additional file 1: Table S1). The resistance threshold was set in accordance with the European Committee on Antimicrobial Susceptibility Testing (https://www.eucast.org/clinical_breakpoints/).

Genomic DNA from each isolate were extracted and purified using a QIAamp DNA Mini Kit (Qiagen, Valencia, CA, USA) according to the manufacturer’s protocol. DNA libraries were prepared using a Nextera XT DNA library preparation kit (Illumina, San Diego, CA, USA). All isolates were sequenced using the Illumina NovaSeq 6000 platform, according to the manufacturer’s instructions. Trimmomatic software version 0.39 was used to filter out the adapter sequence and low-quality bases/reads. A quality assessment of the sequence reads was performed using FastQC version 0.11.9. The clean reads were mapped to the reference strain FA1090 (GenBank accession no. AE004969.1) using BWA MEM [22]. The sequences were uploaded to the National Center for Biotechnology Information for Biotechnology Information Sequence Read Archive (PRJNA560592).

A concatenate superset of SNPs relative to FA1090 was generated as previously described [23]. MEGA 11 [24] was used to constructed maximum-likelihood phylogeny. Multilocus sequence typing (MLST) and *N. gonorrhoeae* sequence typing for antimicrobial resistance (NG-STAR) were performed using gene sequences extracted in silico from the WGS data by mapping clean reads to the reference genome FA1090 (GenBank accession no. AE004969.1) and submitted to the *Neisseria* MLST (http://www.mlst.net/) and NG-STAR (https://ngstar.canada.ca/welcome/home) websites to determine the respective STs. SWISS-MODEL (https://swissmodel.expasy.org/interactive) was used to predict the protein structures of *penA*-64.001 and *penA*-232.001.

## Results and discussion

Twelve ceftriaxone-resistant isolates were isolated from ten hetero- and two homosexual male patients (Additional file 1: Table S1). All cases were first-time gonorrhea infection. They consulted the Sichuan Provincial People’s Hospital in Chengdu, China because of urethral discharge. The current treatment regimen in China suggest an increase of ceftriaxone dose in the treatment from 250 mg in 2014 to 1 g in 2020, which can only temporarily alleviate the gonorrhea treatment with the evolution of gonococcal drug resistance [25]. All patients were intramuscularly administered ceftriaxone (1 g), and none of them come back for a follow-up visit.

Ten isolates (83.3%) were multidrug resistant and were identified with the *penA*-60.001 allele (Additional file 1: Table S1), suggesting that they potentially belong to the FC428 clone. In addition, 75% (9/12) of the isolates showed a typical ceftriaxone-resistant phenotype (MIC ≥ 0.5 mg/L), and 16.7% (2/12) showed an intermediate evaluated azithromycin MIC of 1 mg/L (Additional file 1: Table S1). Interestingly, the MIC values of cefixime was observed to be lower than that of ceftriaxone (Additional file 1: Table S1), which may be due to the inhibitory effect of the novel mutations on cefixime resistance.

Six isolates belonged to ST1903, and were identical to the original FC428 clone [2]. Three and two isolates were assigned to Chinese epidemic clones ST8123 and ST7363, respectively (Additional file 1: Table S1). ST8123 was the predominant ST and a subgroup founder in the GoeBURST analysis in Shenzhen in 2014–2018 [1]. Here, we report three ST8123 isolates that harbored a mosaic *penA*-60.001 allele after this clone first reported in United Kingdom in 2022 [26], suggesting that locally prevalent clones may acquire the mosaic *penA*-60.001 allele and gain ceftriaxone resistance during transmission and expansion.

Seven NG-STAR types were identified with five new profiles (Additional file 1: Table S1). The ten FC428 isolates were assigned to six STs. Briefly, CD19-21 was assigned to ST233, and five isolates belonged to ST1143, which was identical to the previously reported Changsha isolates [27], suggesting further dissemination of ST1143. These results indicate that the FC428 clones were disseminated in distinct regions of China and did not reduce their biological fitness. The four isolates were assigned to four new types: ST4903, ST4904, ST4905, and ST4906 (Additional file 1: Table S1). Interestingly, the remaining CD20-24 and CD20-63 displayed the same novel ST4510 (Additional file 1: Table S1), processing a novel mosaic *penA*-232.001 allele with great similarity to resistant *penA*-64.001 allele, and showing an amino acid identity of 99.3%. Although the MIC of *penA*-232.001 (MIC = 0.25 mg/L) was not enough to be defined as resistance for some recommended regimens [14, 28]. However, except showing a close genetic relatedness to *penA-*64.001 of the old sporadic ceftriaxone-resistant isolate A8806, the *penA*-232.001 also carried the well-known mutations A311V, T483S, and N513Y those were associated with cephalosporin resistance. All mentioned risk factors make it essential to increase awareness of these isolates to prevent the accumulation of resistance during transmission and expansion.

**Fig. 1:**
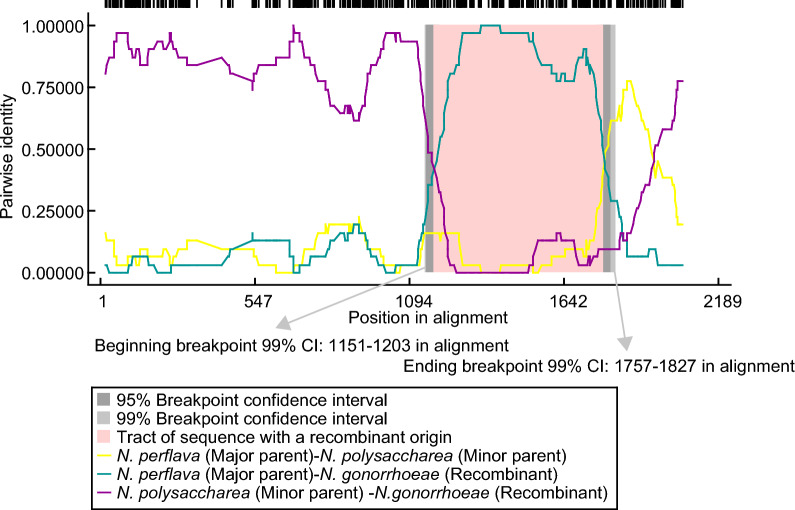
Recombination analysis of *penA*-232.001 gene. An alignment of *N. gonorrhoeae* and commensal *Neisseria* species *penA* sequences were analysed using the RDP4 software. The X-axis and Y-axis represent the position in alignment and pairwise identity, respectively. The yellow line represents the pairwise of *N. perflava* as a major parent, and *N. polysaccharea* as a minor parent. The green line represents the pairwise of *N. perflava* as a major parent, and *N. gonorrhoeae* as a recombinant. The purple line represents the pairwise of *N. polysaccharea* as a minor parent, and *N. gonorrhoeae* as a recombinant

Potential recombinant events in *penA* between *N. gonorrhoeae* and commensal *Neisseria* species were analyzed using recombination detection program (RDP4) [29], using the standard settings with default values. Results demonstrated that *penA*-232.001 (OR125086) had high sequence identity with *Neisseria* species (*N. perflava* (AB904122) and *N. polysaccharea* (NZ_CP031325.1:707543–705798)) and indicated homologous recombination events (Fig. 1), which provide evidence of the possibility that the commensal *Neisseria* species might serve as a reservoir of ceftriaxone resistance-mediating *penA* sequences in clinical gonorrhoeae strains.

**Fig. 2:**
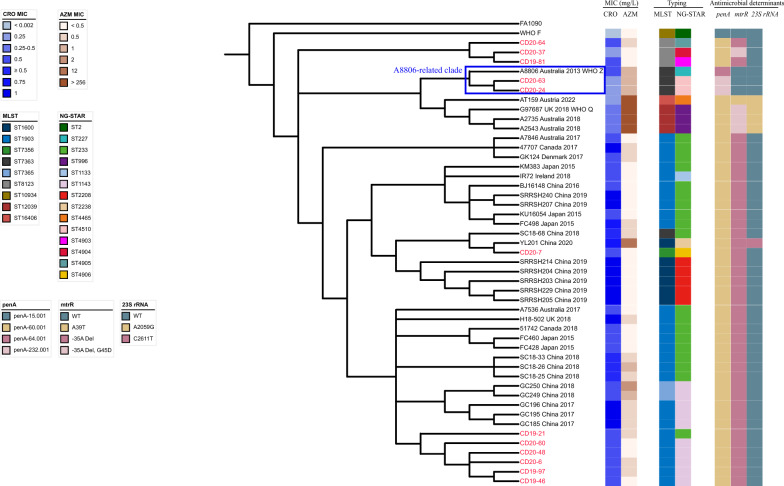
Maximum-likelihood phylogeny of 21,715 SNPs along the whole genome. A total of 34 previously reported FC428-like isolates, the Australian A8806 and WHO-F were included in the phylogenetic analysis. Isolates with red font colour are those characterized in the current study. STs, antimicrobial resistance determinants and antimicrobial susceptibilities are shown. CRO: ceftriaxone, AZM: azithromycin

Maximum-likelihood phylogenetic analysis based on the core-genome alignment of 21,715 SNPs was performed as previously described [23]. These 12 isolates were distributed into four subclades (Fig. 2). The six FC428 isolates clustered with five Changsha isolates and three previously described Chengdu isolates (Fig. 2). CD20-7 clustered with Shenzhen isolate YL201 and the previously reported Chengdu isolate SC18-68. These findings support the separate introduction of FC428 from different regions and the development of subsequent local transmission. Moreover, our study is the second report of FC428 clone in Chengdu since Wang et al*.* reported four FC428 isolates out of 112 *N. gonorrhoeae* isolates in 2018 [13], indicating successful local dissemination of this clone. Notably, CD20-24 and CD20-63 clustered closely with multidrug-resistant A8806, which was initially identified in Australia in 2013 [16]. Interestingly, CD20-24 and CD20-63 had the same phenotypes and MLSTST7363 with A8806 (Fig. 2). Only four substitutions in *penA*-232.001 were found compared to *penA*-64.001 in A8806 (Additional file 1: Fig. S1). Based on reference mapping, CD20-24 and CD20-63 only differed from A8806 by 1582 and 1683 SNPs among the whole genome, respectively. The predicted protein structure of *penA*-64.001 and *penA*-232.001 was shown in Additional file 1: Fig. S2, which reveals the protein fold diversity of the penicillin-binding protein 2 during transmission. It might influence the ceftriaxone binding affinity, however, it warrants further confirmation at the molecular level. Additionally, CD20-24, CD20-63, and A8806 clustered with four recently reported ceftriaxone-resistant and high-level azithromycin-resistant isolates (Fig. 2), suggesting an increasing trend in azithromycin resistance and posing a threat to the effectiveness of dual-antimicrobial therapy. Three of the four cases were associated with travel to Southeast Asia [30, 31], suggesting a circulation in Asia. Unlike the mosaic alleles *penA*-37.001 [32] causing lower biological fitness and limiting further spread [33], the emergence of CD20-24 and CD20-63 provided evidence for the further spread of A8806, indicating a wide spread similarity to that of FC428.

## Conclusion

In conclusion, we identified ten FC428 isolates and characterized new ceftriaxone-resistant isolates in Chengdu between 2019 and 2010. These results suggest that the FC428 clone has further disseminated in China. The emergence of novel isolates and their close genetic relatedness to A8806 suggest a potential resurgence risk and spreading ability of the old ceftriaxone-resistant A8806, which may develop diversity of drug-resistance mechanisms. Our results provide evidence of interspecies recombination of *penA* genes between *N. gonorrhoeae* and commensal *Neisseria species.* (*N. perflava* and *N. polysaccharea*). It is essential to increase awareness of both FC428 and sporadic ceftriaxone-resistance gonococcal isolate A8806, which has the potential to become the next “superbug”. Comprehensive strategies for diagnosis of *N. gonorrhoeae* and accurate detection of antimicrobial resistance (AMR), such as combining standard AMR monitoring methods with molecular markers, can be used in AMR surveillance programs and inform treatments and should be the focus of future research.

### Supplementary Information

**Additional file 1: Table S1.** Antimicrobial susceptibility and molecular characteristic of 12 ceftriaxone resistant isolates from Chengdu, China, 2019-2020. **Fig. S1.** Amino acid alignment of *penA*-64.001 and *penA*-232.001 (from amino acids 275 to 295 of *penA* gene). **Fig.** **S2.** Penicillin-binding protein 2 crystal structure of penicillin-binding protein 2 of **A** *penA*-64.001 and **B** *penA*-232.001.

## Data Availability

Not applicable.

## Abbreviations

*N. gonorrhoeae*: *Neisseria gonorrhoeae*
MIC: Ceftriaxone minimum inhibitory concentration
MLST: Multilocus sequence typing
NG-STAR: *N. gonorrhoeae* Sequence typing for antimicrobial resistance
RDP4: Recombination detection program
*N. perflava*: *Neisseria perflava*
*N. polysaccharea*: *Neisseria polysaccharea*
AMR: Antimicrobial resistance
CRO: Ceftriaxone
AZM: Azithromycin
PEN: Penicillin
CIP: Ciprofloxacin
SPT: Spectinomycin
CFM: Cefixime
