# Supplementary material for: Identification of ceftriaxone-resistant Neisseria gonorrhoeae FC428 clone and isolates harboring a novel mosaic penA gene in Chengdu in 2019–2020
Source: Ann Clin Microbiol Antimicrob. 2023 Aug 17;22:73. doi: 10.1186/s12941-023-00614-x (PMC10436653; doi:10.1186/s12941-023-00614-x)
Supplement: Supplementary file 1 — Additional file 1: Table S1. Antimicrobial susceptibility and molecular characteristic of 12 ceftriaxone resistant isolates from Chengdu, China, 2019-2020. Fig. S1. Amino acid alignment of penA-64.001 and penA-232.001 (from amino acids 275 to 295 of penA gene). Fig. S2. Penicillin-binding protein 2 crystal structure of penicillin-binding protein 2 of A penA-64.001 and B penA-232.001. [file 12941_2023_614_MOESM1_ESM.docx]

**Additional file Material Files**

**Identification of ceftriaxone-resistant *Neisseria gonorrhoeae* FC428 clone isolates harboring a novel mosaic *penA* gene in Chengdu in 2019–2020**

**Additional file Material Files**

**Table S1** Antimicrobial susceptibility and molecular characteristic of 12 ceftriaxone resistant isolates from Chengdu, China, 2019-2020.

**Fig. S1** Amino acid alignment of *penA*-64.001 and *penA*-232.001 (from amino acids 275 to 295 of *penA* gene).

**Fig. S2** Penicillin-binding protein 2 crystal structure of penicillin-binding protein 2 of (A) *penA*-64.001 and (B) *penA*-232.001.

Table S1 Antimicrobial susceptibility and molecular characteristic of 12 ceftriaxone resistant isolates from Chengdu, China, 2019-2020.

| Isolate | Year | Country | Patient Gender | Sexual Orientation | History of Tourism | Sampling Site | NG-STAR | *penA* allele | MIC (mg/L) | | | | | | |
| --- | --- | --- | --- | --- | --- | --- | --- | --- | --- | --- | --- | --- | --- | --- | --- |
|  |  |  |  |  |  |  |  |  | MLST | CRO | AZM | PEN | CIP | SPT | CFM |
| CD19-21 | 2019 | China | Male | Hetero | NO | Urethral | 233 | 60 Mosaic | 1903 | 0.5 | 0.5 | 4 | ≥16 | 16 | 0.25 |
| CD19-46 | 2019 | China | Male | Hetero | NO | Urethral | 1143 | 60 Mosaic | 1903 | 0.5 | 0.125 | ≥8 | ≥16 | 8 | 0.25 |
| CD19-81 | 2019 | China | Male | Hetero | NO | Urethral | 4903* | 60 Mosaic | 8123 | 0.5 | 0.25 | ≥8 | ≥16 | 32 | 0.25 |
| CD19-97 | 2019 | China | Male | Hetero | NO | Urethral | 1143 | 60 Mosaic | 1903 | 0.5 | 0.5 | 4 | ≥16 | 16 | 0.25 |
| CD20-24 | 2020 | China | Male | Hetero | NO | Urethral | 4510* | 232 Mosaic* | 7363 | 0.25 | 1 | 1 | ≥16 | 16 | 0.125 |
| CD20-37 | 2020 | China | Male | Hetero | NO | Urethral | 4904* | 60 Mosaic | 8123 | 0.25 | 0.0625 | ≥8 | 8 | 32 | 0.125 |
| CD20-48 | 2020 | China | Male | Homo | NO | Urethral | 1143 | 60 Mosaic | 1903 | 0.5 | 0.25 | 4 | ≥16 | 16 | 0.125 |
| CD20-6 | 2020 | China | Male | Hetero | NO | Urethral | 1143 | 60 Mosaic | 1903 | 0.5 | 0.5 | 2 | ≥16 | 16 | 0.125 |
| CD20-60 | 2020 | China | Male | Hetero | NO | Urethral | 1143 | 60 Mosaic | 1903 | 0.5 | 0.25 | 2 | 8 | 16 | 0.125 |
| CD20-63 | 2020 | China | Male | Hetero | NO | Urethral | 4510* | 232 Mosaic* | 7363 | 0.25 | 1 | 1 | ≥16 | 16 | 0.0625 |
| CD20-64 | 2020 | China | Male | Homo | NO | Urethral | 4905* | 60 Mosaic | 8123 | 0.5 | 0.5 | ≥8 | ≥16 | 32 | 0.125 |
| CD20-7 | 2020 | China | Male | Hetero | NO | Urethral | 4906* | 60 Mosaic | 7356 | 0.5 | 0.25 | 2 | ≥16 | 16 | 0.125 |

Abbreviations: Abbreviations: CRO: ceftriaxone, AZM: azithromycin; PEN: penicillin; CIP: ciprofloxacin; SPT: Spectinomycin; CFM: cefixime.

Note: STs and alleles followed by an asterisk were new STs and alleles submitted in the current study.


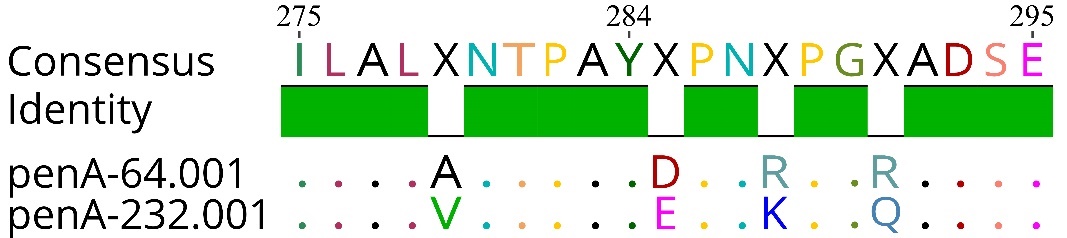


**Fig. S1** Amino acid alignment of *penA*-64.001 and *penA*-232.001 (from amino acids 275–295 of *penA* gene). Substitutions: A279V, D285E, R288K, and R291Q. Identity = 99.3% (578/582).


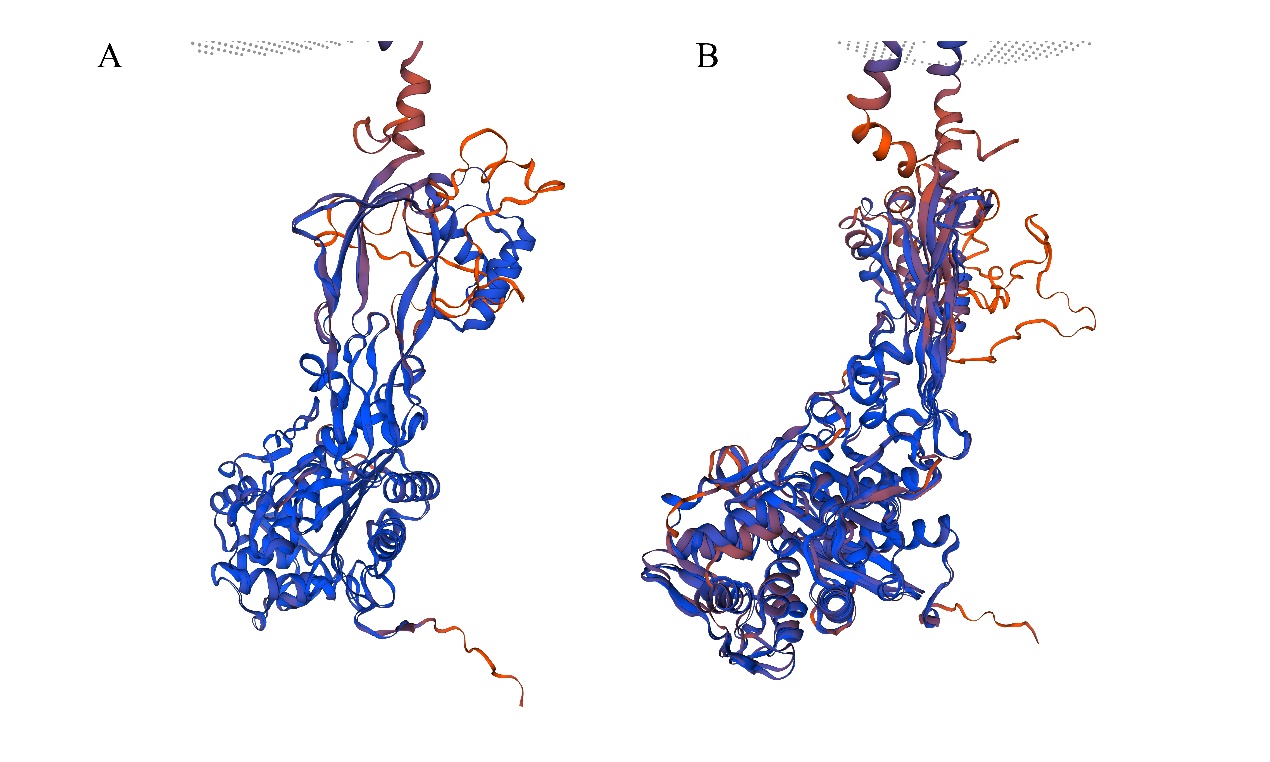


**Fig. S2** Penicillin-binding protein 2 crystal structure of penicillin-binding protein 2 of (A) *penA*-64.001 and (B) *penA*-232.001.
